# Isoflurane Preconditioning at Clinically Relevant Doses Induce Protective Effects of Heme Oxygenase-1 on Hepatic Ischemia Reperfusion in Rats

**DOI:** 10.1186/1471-230X-11-31

**Published:** 2011-03-31

**Authors:** Xin Lv, Liqun Yang, Kunming Tao, Yantao Liu, Tian Yang, Guozhong Chen, Weifeng Yu, Hao Lv, Feixiang Wu

**Affiliations:** 1 Department of Anesthesia and Intensive Care, Eastern Hepatobiliary Surgery Hospital, the Second Military Medical University, 225 Changhai Road, Shanghai 200438, PR China; 2 Department of Anesthesiology, Shanghai Pneumology Hospital, Tongji University School of Medicine, Shanghai, 200433, PR China; 3 Department of Anesthesia, Fuzhou general hospital affiliated to Nan Jing Military District. Fuzhou, 350000, PR China

## Abstract

**Background:**

Activation of heme oxygenase-1 (HO-1) has been proved to reduce damages to the liver in ischemia reperfusion injury. The objective of present study was to determine whether clinic relevant doses of isoflurane treatment could be sufficient to activate HO-1 inducing, which confers protective effect against hepatic ischemia-reperfusion injury.

**Methods:**

The hepatic artery and portal vein to the left and the median liver lobes of forty male Sprague-Dawley rats were occluded for 60 minutes. Reperfusion was allowed for 4 hours before the animal subjects were sacrificed. Six groups (n = 12) were included in the study. A negative control group received sham operation and positive control group a standard ischemia-reperfusion regimen. The third group was pretreated with isoflurane prior to the ischemia-reperfusion. The fourth group received an HO-1 inhibitor zinc protoporphyrin (Znpp) prior to the isoflurane pretreatment and the ischemia-reperfusion. The fifth group received Znpp alone before ischemia-reperfusion procedure, and the sixth group was administrated with a HO-1 inducer hemin prior to IR. HO-1 in the liver was measured using an enzymatic activity assay, a Western blot analysis, as well as immunohistochemical method. Extent of liver damage was estimated by determination of the serum transaminases, liver lipid peroxidation and hepatic histology. Infiltration of the liver by neutrophils was measured using a myeloperoxidase activity assay. TNFα mRNA in the liver was measured using RT-PCR.

**Results:**

Isoflurane pretreatment significantly attenuated the hepatic injuries and inflammatory responses caused by the ischemia reperfusion. Selectively inhibiting HO-1 with ZnPP completed blocked the protective effects of isoflurane. Inducing HO-1 with hemin alone produced protective effects similar in magnitude to that of isoflurane.

**Conclusions:**

Clinic relevant doses of isoflurane attenuate ischemia reperfusion injury in rats by increasing the HO-1 expression and activity.

## Background

The heme oxygenase (HO) has been shown to limit reperfusion injury after experimental systemic and regional hepatic ischemia[1]. Heme oxygenase 1(HO-1) is the only inducible form of HO family and its gene expression is up-regulated in many tissues exposed to a wide spectrum of noxious stimuli, including physical (irradiation, hyperthermia, etc.), chemical (heavy metals, carbon tetrachloride, etc.), and physiological (hypoxia, endotoxemia, etc.) insults. HO-1 catalyzes the oxidation of heme to biliverdin-IXa, iron, and carbon monoxide, which exerts its antioxidative, anti-inflammatory, antiapoptotic, and vasodilatory effects. Activation of HO-1 has also been shown to reduce damages to the liver caused by a multitude of factors, including hemorrhagic shock, endotoxemia, acetaminophen, and IR[2-10]. Therefore, HO-1 appears to be a promising candidate for minimizing the damage after hepatic IR.

Schmidt et al.[11] have confirmed that pretreatment with isoflurane (ISO) induces hepatic HO-1 expression and thereby protects rat lives from IR injury. However, a rather long ISO pretreatment time of 5.5 hours and a high dose of ISO (2.4 MAC) was required in that study. Whether shorter time intervals or lower concentrations of ISO treatment could be sufficient to activate HO-1 and confer protective effect still needs to be evaluated.

In the current study, a pharmacological approach was used to explore there was a cause-effect relationship between HO-1 induction and cellular protection in a rat model of partial hepatic ischemia followed by reperfusion. Specifically, effects of the selective HO-1 inhibitor zinc protoporphyrin (Znpp) and the selective HO-1 inducer hemin, and their interaction with isoflurane pretreatment were also examined under hepatic IR process.

## Methods

### 1 Experimental animals

Male Sprague-Dawley rats (8-10 weeks, 180-220 g) from the Experimental Animal Center, the Chinese National Institute of Medicine (Shanghai, China) were used. Animals were housed in an air-conditioned room at a temperature of 22-25°C, with unlimited access to tap-water and standard rat chow. Food was removed from the cages at 12 hours prior to the experiments. The experimental protocol was approved by the Animal Care and Scientific Committee of the Second Military Medical University, Shanghai, China.

### 2 Partial hepatic ischemia and reperfusion

A model of segmental (70%) hepatic ischemia was used as previously described[12]. After laparotomy with a midline incision under anesthesia with sodium pentobarbital (40 mg/kg, i.p.), the ligaments around the liver were located and disconnected. The hepatic artery, portal vein, and bile duct to the left and median hepatic lobes were carefully revealed, and occluded with an atraumatic vascular clamp. The clamp was removed 60 minutes afterward to allow reperfusion. The incision was closed with sutures during the reperfusion. Body temperature was maintained at 36-37°C (rectal) by a heating lamp throughout the entire procedure. Animal subjects were sacrificed 4 hours after reperfusion started.

### 3 Isoflurane treatment

A home-made plexiglass box was used to deliver isoflurane (Abbott Laboratories. Abbott Park, Illinois). Dimensions of the box were 50 × 15 × 15 cm^3^, with in- and out-flow at the opposite long ends. Air sample was taken from a hole adjacent to the air outlet. Two holes (10 cm in diameter) sealed with rubber gloves on a side panel were used for maneuvering the rats. Temperature was maintained at 35-37°C using light bulbs and partial pressure of CO_2 _was maintained at < 0.05% atm with natrica calx. An air/oxygen (35%) mixed gas was delivered at a flow rate of 3 L/min.

Isoflurane was delivered with the air/oxygen mixture using an agent-specific vaporizer (Datex-Ohmeda; Madison, WI). Rats were placed into the box 15 minutes after the isoflurane concentration reached a steady level of 1.4%. Concentrations of isoflurane and oxygen were measured continuously using a Datex respiratory gas analyzer (Datex Instrumentarium; Helsinki, Finland).

### 4 Experimental groups

In addition to a sham operation group, the study was comprised of 5 more groups (n = 12 in each group). The first group received a standard ischemia-reperfusion procedure, and served as a positive control. The second group was pretreated with 1.4% isoflurane for 30 minutes prior to the ischemia-reperfusion regimen. The treatment to the third group was identical to the isoflurane group, except that the animal subjects received an intraperitoneal injection (i.p.) of selective HO-1 inhibitor zinc protoporphyrin (Znpp; 25 μmol/kg; Sigma Chemicals, Germany) at 1 hour prior to isoflurane. Animals in the fourth group were injected 25 μmol/kg of Znpp i.p. at 1.5 hour before IR. The fifth group received an injection of the HO-1 inductor hemin (50 mg/kg; Sigma, St. Louis, MO) i.p. 24 hours prior to IR. All these animals except that in sham group subsequently underwent the same IR procedure as the first group.

### 5 Sample collections

Four hours after the reperfusion started, rats received an additional dose of sodium pentobarbital (40 mg/kg, i.p.), and were sacrificed by severing the abdominal aorta. Serum were collected and stored at -20°C. The median lobe of the liver was fixed with 10% formalin and imbedded with paraffin. The left lobe were rapidly frozen in liquid nitrogen and stored at -80°C.

### 6 Hepatic HO-1 activity assay

HO-1 activity was assessed using a method reported previously[13-16]. Liver tissue was homogenized in 4 volumes of a phosphate buffer (0.1 mM KH_2_PO_4_; pH 7.4) briefly using an ultrasonic homogenizer (Ultraturrax; Janke and Kunkel, Staufen, Germany). The homogenate was centrifuged at 10,000 g at 4°C for 20 minutes. The supernatant was used for the measurement of HO-1 activity. Final concentration of the reaction buffer consisted of 0.8 mM NADPH, 2 mM glucose-6-phosphate, 0.002 U/mL glucose-6-phosphate dehydrogenase, and 20 μM hemin. The reaction volume was 500 μl, containing 200 μl supernatant and 100 μl liver cytosol from normal rat as a source of biliverdin reductase. Incubation was carried out in a completely dark room for 60 minutes at 37°C. The reaction was terminated by immersion of the test tubes in an ice-bath. For extraction of bilirubin, the tubes were mixed thoroughly and it was followed by centrifugation of the tubes at 13,000 rpm for 5 minutes. The chloroform layers were scanned on a spectrophotometer at 464 nm minus the background at 530 nm. Enzymatic activity was expressed as pmol/mg protein/hr.

### 7 Western blot analysis of HO-1

Liver samples were homogenized in 5 volumes of 10 mM Tris buffer (pH 7.4) containing 1% Triton X-100, 0.5% NP-40, 1 mM EGTA, 1 mM EDTA, 150 mM NaCl and 1 mM PMSF. The protein concentration in liver homogenate was determined using a Bradford assay (Nanjing Jiancheng Bioengineering Institute, Nanjing, China). The homogenate was centrifuged at 16,500 g for 15 minutes. Supernatant containing 30 μg protein was subjected to 12% SDS-PAGE. Separated protein bands were transferred onto an nitrocellulose filter. The filter was blocked with 3% non-fat milk, and incubated with a rabbit-anti-mouse multiclonal antibody against the HO-1 (dilution 1:2000; ABCAM; Cambridge, UK) for 2 hours. After washing with TBST, the filter was incubated with the secondary antibody (peroxidase labeled sheep-anti-rabbit IgG) at a dilution of 1:2000 for 1 hour. Chemoluminescence agent A was mixed with B at equal volume and evenly smeared onto the filter. The chemiluminescence signals captured by exposure to an X-ray film were digitized and then analyzed by a Chemi-Smart 3000 (Vilber Lourmat; Marne-la-Vallée, France).

### 8 Serum transaminases, hepatic myeloperoxidase, malondialdehyde and TNFα mRNA real-time RT-PCR

Serum alanine aminotransferase (ALT) and aspartate aminotransferase(AST) levels were determined with an autoanalyzer (Model 7600, Hitachi Co.; Tokyo, Japan). Hepatic tissue of weight of 100 mg was homogenized in 900 μl phosphate buffer (pH 7.4). The homogenate was centrifuged at 4,000 g for 10 minutes. Hepatic tissue change of myeloperoxidase (MPO) and malondialdehyde (MDA) were assessed by biochemical kits purchased commercially (Nan-Jing Jiancheng Biochemicals Ltd, China). The detection ranges were 0-100 U.ml-1 with a sensitivity of 1 U/ml for MPO, and 0-200 mmol/l with a sensitivity of 1 mmol/l for MDA.

Total RNA was extracted from liver using the Trizol reagent (Takara Bio Inc, Japan) as described in the manufacturer's instructions. TNF-α mRNA were quantified in duplicate by SYBR green two-step, real-time RT-PCR. Briefly, following removal of potentially contaminating DNA using DNase I (Invitrogen, Carlsbad, CA), 1 μg of RNA from each sample was used for reverse transcription with oligo dT (Invitrogen, Carlsbad, CA) and Superscript II (Invitrogen, Carlsbad, CA) to generate first-strand cDNA. The PCR reaction mixture was prepared using SYBR green PCR Master Mix (Applied Biosystems, Foster City, CA) using primers as follows: TNF-α 5'-CCCGGAATGTCGATGCCTGAGTG-3', and 5'-CGCCCCGGCCTTCCAAATAAAT-3'; Thermal cycling conditions were 10 minutes, at 95 ° C, which was followed by 40 cycles of 95°C for 15 seconds and 60°C for 1 minute on a sequence detection system (ABI PRISM 7000; Applied Biosystems, Foster City, CA). Each expression gene was normalized with GAPDH mRNA using a Delta-Delta CT method.

### 9 Histology examination and immunohistology of HO-1

All the histopathology and immunohistologic examination were performed by a blinded pathologist to identify underlying necrosis and apoptosis status or other types of injury. Liver samples were excised from the anterior edge of the left lobe 120 minutes after reperfusion. Small portions (0.5 cm × 0.5 cm) were fixed immediately in 10% buffered par formaldehyde (pH 7.2) and embedded in paraffin. These portions were cut into 4 μm thick sections and stained with hematoxylin and eosin (H&E). High-powered microscopy (1 × 200) was used to examine theses sections for the following signs of liver injury: condensation of nuclei (nuclear pyknosis), loss of hepatocellular borders, or areas of necrosis, A score determined by dividing the measured necrotic area by the total area of the field using Image-Pro-Plus® Software (Media Cybernetics Inc, Bethesda, MD) was obtained. HO-1 immuno-reactivity was examined using an Elivision^TM ^Plus Broad Spectrum kit (Fujian Maixin Biological Technology; Fujian, China). Tissue sections were incubated at room temperature with 3% hydrogen peroxide to deactivate endogenous peroxidases after deparaffinage and re-hydration. Sections were dipped into an antigen retrieval buffer (10 mM sodium citrate; pH 6.0) and underwent warm retrieval in a microwave oven at 95-100°C for 10 minutes. Sections were incubated with a rabbit-anti-mouse HO-1 antibody (dilution 1:100; ABCAM; Cambridge, UK) at room temperature for one hour. After washing with PBS, a polymer enhancer and an anti-rabbit IgG labelled with horseradish peroxidase was applied. HO-1 was visualized as buffy granules in the cytoplasma using a DAB kit (Fujian Maixin Biological Technology).

### 10 Statistical analyses

Data analysis was performed with a personal computer with statistical software package (Prism version 4.0; Graph-Pad Software, San Diego, CA). Data are expressed as mean ± SD. One way analysis of variance and the Student-Newman-Keuls q test were used to compare values among all groups. Serum enzymes and peroxidation and TNF alpha mRNA variables were analyzed using one way analysis of variance with Student-Newman-Keuls test. Statistical differences were considered significant if the *P *value was less than 0.05. All *P *values were results of two-sided tests.

## Results

### 1 Effects of isoflurane preconditioning on the hepatic HO-1ativity and expression

**Figure 1:**
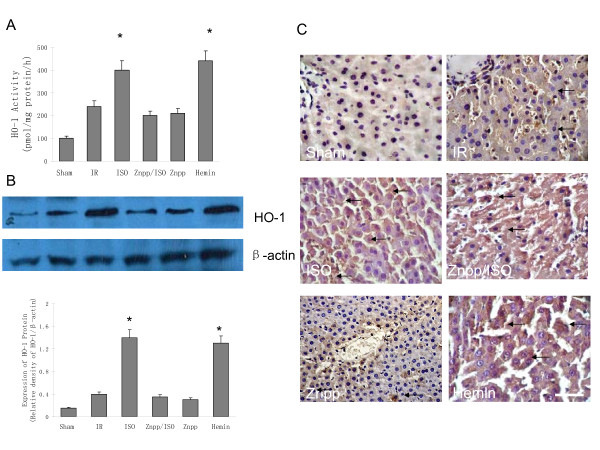
**Effect of isoflurane preconditioning on liver heme oxygenase-1(HO-1) after ischemia reperfusion injury (IR) (n = 12 each group)**. (A) The HO-1 activity in the liver. Both isoflurane and hemin pretreatment augmented the HO-1 activity response, while administration of the HO-1 inhibitor zinc protoporphyrin (Znpp), prior to the isoflurane pretreatment or Znpp alone significantly decreased the HO-1 activity (p < 0.05, *vs*. IR). (B) Western blot analysis of HO-1in liver. Western blotting showed an increase in HO-1 protein expression in the groups of isoflurane and hemin (* P < 0.05 *vs*. IR). (C) Immunohistochemical detection of HO-1 in the liver. Hepatic HO-1 positive cells were defined as stained with brown in cytoplasm (black arrows). There was an increase in HO-1 expression in isoflurane and hemin pretreated groups compared in IR, it also showed a decrease in HO-1protein expression in both Znpp pretreated groups compared with isoflurane group (Magnification: 400×. Scale bar = 100 μm).

The ischemia/reperfusion regimen significantly increased the hepatic HO-1 activity (Figure 1A). Isoflurane pretreatment augmented the HO-1 activity response induced by the ischemia/reperfusion. Administration of the HO-1 inhibitor Znpp, prior to the isoflurane pretreatment or pretreatment of Znpp alone significantly reduced the HO-1 activity (p < 0.05, compared to IR). Pretreatment with the HO-1 inducer hemin increased the hepatic HO-1 activity to a level similar to that in the isoflurane group.

Changes in the HO-1 protein level, as determined by the Western blot analysis ((Figure 1B). HO-1 was barely detectable under the baseline condition. IR increased the amount of HO-1 significantly. Isoflurane or hemin pretreatment further increased the expression of HO-1, while no difference in the expression of HO-1 protein level in Znpp/isoflurane and Znpp groups could be found when compared with IR group. Results from the immunohistology were also in line with the changes in enzymatic activity as shown in figure 1C. Very few HO-1 positive cells were identified in the sham controls. IR increased the number of HO-1 positive cells, while isoflurane or hemin substantially increased the number of HO-1 positive cells in comparison to the IR group, and Znpp decreased HO-1 expression when compared with isoflurane group.

### 2 Effects of isoflurane preconditioning on hepatic injury after ischemia reperfusion procedure

**Figure 2:**
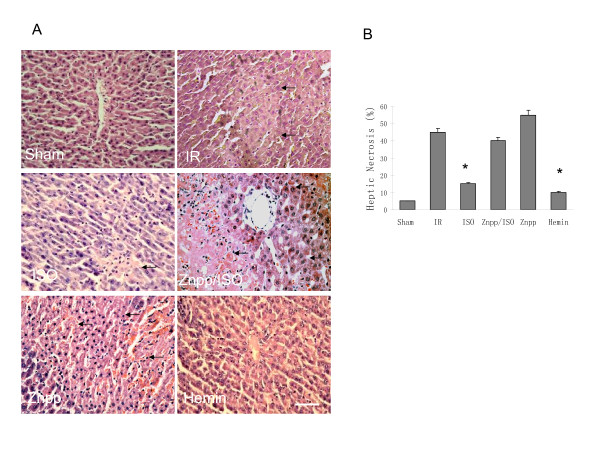
**Effects of isoflurane preconditioning on hepatic histology after ischemia reperfusion (IR) procedure (n = 12 in each group)**. (A) Hepatic tissue histological changes were processed with H&E staining for light microscopy examination. Photograph depicted typical pattern of focal necrosis (black arrows) after ischemic degeneration of hepatocytes around the central venous area. More seriously necrosis was shown in both zinc protoporphyrin (Znpp) pretreated and IR groups compared in sham group. Magnification: 200 × Scale bar = 200 μm. (B) There was no obvious necrosis in sham group. Areas of necrosis were significantly lower in isoflurane and hemin pretreated groups than in IR group (* P < 0.05 *vs*. IR).

The ischemia/reperfusion regimen significantly increased the serum transaminases, hepatic TNFα mRNA level, myeloperoxidase and malonaldehyde activity (shown in table 1). All these changes were attenuated significantly by the pretreatment with isoflurane and hemin. Administration of Znpp completed reversed these effects. Four hours after reperfusion, the liver histology of isoflurane and hemin pretreated groups exhibited fewer and smaller areas of necrosis and structural derangement around the central vein as compared to the IR group, while there were no obvious histological difference noticed in Znpp/isoflurane and Znpp groups when compared to the IR((Figure 2).

**Table 1:** Serum aminotransferases, liver lipid peroxdation, myeloperoxidase activity and TNFα mRNA when pretreated with isoflurane

	Sham	IR	ISO	Znpp/ISO	Znpp	Hemin
ALT (IU.l^-1^)	39 ± 14*	3336 ± 393	2787 ± 209*	3677 ± 249	3106 ± 327	2290 ± 334*
AST (IU.l^-1^)	31 ± 25*	3792 ± 326	2867 ± 475*	4149 ± 401	3904 ± 414	2149 ± 509*
Hepatic TNFα mRNA (fold of Sham)	-	12.8 ± 7.2	6.7 ± 3.5*	14.5 ± 7.8	13.6 ± 6.9	7.5 ± 4.9*
MDA (μmol.l^-1^)	4.8 ± 1.6*	29.6 ± 7.2	13.1 ± 1.8*	39.4 ± 10.6	37.6 ± 8.8	19.8 ± 4.7*
MPO (U.g tissue^-1^)	1.5 ± 0.3*	3.5 ± 0.3	2.2 ± 0.4*	3.7 ± 0.5	4.1 ± 0.7	2.0 ± 0.3*

* P < 0.05 *vs*. IR group value. Values are mean ± SD of 12 animals in each group.

IR = ischemia reperfusion injury; ISO = isoflurane; Znpp = zinc protoporphyrin; ALT = alanine aminotransferase; AST = aspartate aminotransferase; TNFα = tumor necrosis factor α; MDA = malondialdehyde; MPO = myeloperoxidase

## Discussion

Results from the current study demonstrated that 1.4% ISO for 30 minutes pretreatment before ischemia significantly increased HO-1 activity in the liver. The increase in enzymatic activity was associated with an increase in the HO-1 protein levels. We also found that increased HO-1 activity and expression by isoflurane was associated with lower levels of aminotransferases and TNFα mRNA, decreased lipid peroxidation and myeloperoxidase activity in the liver.

HO-1 expression could be induced including solitude hypoxia or hyperoxia. Much work has been done and aimed to assess whether hypoxia alone could cause any significant induction of HO-1 or not. [11,14-19] Yamasaki's [19] study in which used low concentration of isoflurane (0.42%) to induce HO-1 in rats' hypoxia model, finding isoflurane-hypoxia treatment caused a minor increase at 6-8 hours. In order to exclude these two possible factors, we used a normal oxygen concentration of 35% which was also in according with clinical application. We also increased the concentration of isoflurane in order to shorten the time of isoflurane treatment. Thus, effects of isoflurane might indeed be clinically relevant. Furthermore, using the inhibitor of HO-1 Znpp, could completely blocked the increase of HO-1 activity and expression when compared with isoflurane pretreatment group. The effects of isoflurane on all measures of liver damage were also completely abolished by Znpp, which strongly suggested that the protective effects of isoflurane were mediated by an increase in HO-1 activity and expression. Effects of pretreatment with the HO-1 inducer hemin alone produced beneficial effects similar to isoflurane, further indicating a pivotal role of HO-1 in the cytoprotective effects under IR.

HO is an almost ubiquitous enzyme that catalyzes the breakdown of heme to equimolar amount of biliverdin, carbon monoxide (CO) and ferrous iron. Among the 3 mammalian isoforms of HO, only the HO-1 is inducible. HO-1 is a major protective mechanism that responds to a wide selection of noxious stimuli, and most notably, oxidative and inflammatory tissue damages[4]. Activation of HO-1 has been shown to minimize the damage after ischemia followed by reperfusion in a number of organ systems[20,21]. In general, IR damage is caused primarily by free radicals. Correspondingly, increased production of biliverdin as a scavenger for free radicals is considered the most important mechanism by which HO-1 counteracts the IR damage[22]. In this particular study, we did not examine the specific role of biliverdin. However many previous studies have demonstrated the importance of this endogenous anti-oxidant.

In addition to the formation of free radicals, IR damage in the liver consists of two additional factors, namely micro-circulation and inflammatory response. We did not directly examine micro-circulation in this study[23,24]. Microscopic examination of hepatic slices, however, indicated that improved micro-circulation might be one of the mechanisms through which HO-1 activation alleviates the IR damage. Thus, the degree of sinosoidal congestion was correlated negatively with HO-1 activity and expression. More specifically, sinosoidal congestion caused by the IR was significantly alleviated by isoflurane and the HO-1 inducer hemin. [25,26] Also, effects of isoflurane were blocked by Znpp. These results were circumstantial in nature, but did point towards a potential involvement of micro-circulation improvement via CO.

The inflammatory response serves an important function of homeostasis after the IR. Ischemia of the liver invariably leads to an influx of bacteria and associated toxins upon reperfusion because of the strategic localization of the liver in blood supply to the entire gastrointestinal tract. [23,25] The inflammatory response after the IR is initiated in the Kupffer cells. Activated Kupffer cells release massive amount of free radicals and pro-inflammatory cytokines. The resulting inflammatory response serves to prevent the bacteria and their products from entering the systemic circulation after the IR. However, convincing evidence has demonstrated that the massive inflammatory response is also a major cause of hepatic injuries after the IR[4,26].

The fact that TNFα in the liver increased significantly after the IR in our experiments suggested that the liver damage after the IR is indeed partially mediated by the inflammatory responses. Infiltration of the liver by neutrophils further supported a critical role of inflammation in the IR injuries. [27] Considering the aforementioned actions of CO in regulating inflammatory responses,[28] these results strongly suggested that the anti-inflammatory action of increased production of CO is a major contributor to the protective action of HO-1. [29]

Based on the observations that pretreatment with low doses of pro-inflammatory cytokines prior to the IR prevents activity burst of Kupffer cells, it is hypothesized that isoflurane may counteract the IR damage through the same mechanism as preconditioning[30,31]. Thus, modest activation of Kupffer cells prior to the IR creates a condition that prevents further activation of Kupffer cells when IR insult actually happens[32]. This hypothesis seems plausible. However, whether preconditioning indeed occurs *in vivo *remains controversial[33]. Experiments addressing this possibility are currently being conducted in this laboratory to test the pre-conditioning hypothesis.

## Conclusions

In summary, our results confirmed that approximately 1 MAC (1.4%) of isoflurane could enough protecting the liver from the IR injuries; and increase the activity and the expression of HO-1 in hepatocytes. This study also demonstrated that the protective effects of HO-1 against the liver IR damage are mediated by the activation of HO-1. Based on these findings that HO-1 is believed to be, a promising molecular target in developing novel treatment and prevention for hepatic IR injuries.

## Competing interests

The authors declare that they have no competing interests.

## Authors' contributions

XL: data mining and analysis in fulfillment of his MD thesis; LY: ideal, experimental design and part of animal studies; KT and TY: amendment experiment and malondialdehyde assessment; YL: conception and help with statistics; WY and GC: conception writing of the paper and part of animal studies; HL & FW: part of animal studies. All authors read and approve the final version of the manuscript.

## Pre-publication history

The pre-publication history for this paper can be accessed here:

http://www.biomedcentral.com/1471-230X/11/31/prepub
